# IL-33 Attenuates Anoxia/Reoxygenation-Induced Cardiomyocyte Apoptosis by Inhibition of PKCβ/JNK Pathway

**DOI:** 10.1371/journal.pone.0056089

**Published:** 2013-02-14

**Authors:** Tao Rui, Qizhu Tang

**Affiliations:** 1 Division of Cardiology, Department of Medicine, Renmin Hospital of Wuhan University, Wuhan, Hubei Province, People’s Republic of China; 2 Division of Cardiology, Department of Medicine, The Affiliated People’s Hospital of Jiangsu University, Zhenjiang, Jiangsu Province, People’s Republic of China; 3 Critical Illness Research, Lawson Health Research Institute, London, Ontario, Canada; 4 Department of Medicine, Schulich School of Medicine and Dentistry, University of Western Ontario, London, Ontario, Canada; Osaka University Graduate School of Medicine, Japan

## Abstract

**Background:**

Interleukin-33 (IL-33) is a new member of the IL-1 cytokine family. The objectives of present study are to assess whether IL-33 can protect cardiomyocytes from anoxia/reoxygenation (A/R)-induced injury and the mechanism involved in the protection.

**Methods:**

Cardiomyocytes derived from either wild type or JNK1^−/−^ mice were challenged with an A/R with or without IL-33. Myocyte apoptosis was assessed by measuring caspase 3 activity, fragmented DNA and TUNEL staining. In addition, cardiomyocyte oxidative stress was assessed by measuring DHR123 oxidation; PKCβII and JNK phosphorylation were assessed with Western blot.

**Results:**

Challenge of cardiomyocytes with an A/R resulted in cardiomyocyte oxidative stress, PKCβII and JNK phosphorylation, and myocyte apoptosis. Treatment of the cardiomyocytes with IL-33 attenuated the A/R-induced myocyte oxidative stress, prevented PKCβII and JNK phosphorylation and attenuated the A/R-induced myocyte apoptosis. The protective effect of the IL-33 did not show in cardiac myocytes with siRNA specific to PKCβII or myocytes deficient in JNK1. Inhibition of PKCβII prevented the A/R-induced JNK phosphorylation, but inhibition of JNK1 showed no effect on A/R-induced PKCβII phosphorylation.

**Conclusions:**

Our results indicate that IL-33 prevents the A/R-induced myocyte apoptosis through inhibition of PKCβ/JNK pathway.

## Introduction

Cardiovascular diseases remain the number one killer in Western society. Coronary artery disease which is associated with myocardial ischemia accounts for 1 in 6 of deaths in the United States [1]. Reperfusion approach is the most effective means of rescuing the ischemic myocardium, however, reperfusion itself can cause injury to the myocardium and initiate myocardial apoptosis [2]. To date, the mechanism involved in the reperfusion-induced myocardial injury and apoptosis is still not fully understood, thus, lack of effective strategy to prevent the reperfusion-induced injury.

Reperfusion of ischemic myocardium can activate apoptotic pathways leading to the death of cardiomyocytes that were viable just before reperfusion [3]. Apoptosis is a tightly regulated energy-dependent process. Common biochemical features of apoptosis include internucleosomal DNA fragmentation. Fragmentation of DNA to 180 to 200 base pairs by DNAses produces a ladder pattern after electrophoresis. The DNAse responsible for DNA cleavage resides in the cytoplasm in inactive form in normal cells. Upon pro-apoptotic stimulus, DNAse becomes activated by action of caspases. Specifically, caspase-3 has been found to play a key role in regulating apoptosis where its activation lead to cell death and its inhibition lead to cell survival [4].

c-Jun N-terminal kinase (JNK) is an important member of the mitogen-activated protein kinase (MAPK) family. It plays a pivotal roles in regulation of cell function and is readily to be activated by many environmental stimuli including myocardial reperfusion [5]. As a pro-apoptotic kinase, activation of JNK is believed to be important in the induction of cardiomyocyte apoptosis in various pathologies [5]; [6]. The main mechanism of JNK activation is the cell stress including the reactive oxygen species (ROS). However, the exact intracellular signaling pathway involved in the activation of JNK is still not clear.

Protein kinase C (PKC) is another important kinase by which the cell functions are regulated. It is a serine/threonine protein kinase family which has at least 11 isoforms that function in different biological systems. PKCβ is one of conventional (c-PKC) isoforms [7]. It has been reported that I/R challenge results in activation of the PKCβ [8]. Either pharmacological or genetically inhibition of PKCβ can attenuate the I/R-induced tissue injury indicating that PKCβ plays a pivotal role in the I/R-induced tissue injury [8]. Since PKCβ is a key trigger to the activation of MAP kinase JNK [9]; [10]. Thus, there is a possibility that activation of PKCβ/JNK pathway could contribute to the I/R-induced myocardial apoptosis.

Interleukin-33 (IL-33) is a 30 kD protein and is a new member of the IL-1 cytokine family. It plays its biological roles by interacting with ST-2L receptor. IL-33 is broadly expressed in many tissues including the heart [11]; [12]. Previous studies have demonstrated that IL-33 has beneficial actions on various cardiovascular pathologies [11]. It has been reported IL-33 can 1) prevent cardiomyocyte apoptosis induced by hypoxia *in vitro* [13] and 2) attenuates myocardial infarction, improves cardiac function and survival after I/R *in vivo* [13]. We have previously demonstrated that diabetes mellitus down-regulation of myocardial IL-33 which results in increase the vulnerability of the diabetic myocardium to ischemia/reperfusion-induced injury [14]. The study further supports the contention that IL-33 is beneficial to the myocardium. However, the exact mechanism involved in the myocardial protection by the IL-33 is not clear.

In the present study, we further confirmed the beneficial effect of IL-33 in preventing anoxia/reoxygenation (A/R), an *in vitro* count part to I/R *in vivo*, induced myocyte apoptosis and studied the mechanism involved in the protective action of IL-33 by using cultured cardiomyocytes. We demonstrated that 1) A/R-induced myocyte apoptosis is attenuated by exogenous IL-33; 2) A/R challenge to myocytes resulted in increase in PKCβII phosphorylation which is prevented by the IL-33 and inhibition of PKCβII with siRNA specific to PKCβII attenuated the A/R-induced myocyte apoptosis; 3) A/R challenge to myocytes led to increase in JNK phosphorylation which was also prevented by the IL-33 and genetically inhibition of JNK1 diminished the A/R-induced myocyte apoptosis; 4) IL-33 attenuated myocyte oxidative stress after A/R; inhibition of PKCβII prevented the A/R-induced JNK phosphorylation, while inhibition of JNK1 showed no effect on A/R-induced PKCβII phosphorylation.

## Materials and Methods

### Mice

C57BL/6 mice were obtained from Charles River Canada (St. Constant, PQ, Canada) and JNK1^−/−^ mice (C57BL/6 background) were obtained from Jackson laboratories (Bar Harbor, ME, USA). The mice were housed in Victoria Research Laboratory Vivarium Service with a 12-hour light/dark cycle and free access to rodent chow and tap water. Breeding pairs were set up to generate the neonatal mice as a source for isolation of cardiac myocytes. The investigation conforms to the *Guide for the Care and Use of Laboratory Animals* published by the US National Institutes of Health (NIH Publication No. 85-23, revised 1996). The experimental protocols were approved by the University of Western Ontario Animal Care and Use Committee (Protocol No. 2008-053).

### Cardiomyocytes

Cardiomyocytes were isolated from neonatal mouse hearts as previously described [6]. Briefly, the harvested hearts were minced, digested with Collagenase II (160 U/ml, Worthington Biochemical Corp, Lakewood, NJ, USA), washed, and the cells suspended in M199 supplemented with 10% fetal calf serum (FCS). The myocytes were enriched by a preplating approach to remove contaminating cells (fibroblasts and endothelial cells readily adhere, while myocytes do not). The non-adherent cells were removed and cultured in M199 supplemented with 10% FCS. After 48 hrs in culture, the cells had formed a confluent monolayer consisting of 95% myocytes beating in synchrony.

### Anoxia/reoxygenation (A/R) Model

As *in vitro* correlate to ischemia/reperfusion *in vivo*, cardiomyocytes were exposed to an A/R as described previously [6]. Briefly, the myocyte monolayers were exposed to an anoxic chamber for 30 min followed by reoxygenation for up to 24 hrs. The control cardiac myocytes were exposed to normoxia (normoxia/reoxygenation, N/R). In some experiments, recombinant IL-33 (2.5–5 ng/ml) was given to myocytes while they were challenged with A/R.

### Oxidant Stress

Cardiomyocyte oxidative stress was assessed by measuring the oxidation of intracellular dihydrorhodamine 123 (DHR 123; Molecular Probe), an oxidant-sensitive fluorochrome, as described previously [15]. Briefly, the cells were treated with DHR 123 (10 µmol/L) for 30 min before being challenged with A/R or N/R. Thirty min after reoxygenation, the cells were washed with PBS, lysed, and DHR 123 oxidation was assessed spectrophotometrically at excitation and emission wavelengths of 502 and 523 nm, respectively.

### JNK and PKCβ Phosphorylation

Lysates of cardiomyocytes were resolved on 10% SDS-PAGE and transferred to PVDF membranes. Blocked (5% non-fat milk) membranes were used for detection of phosphorylated and total JNK or PKCβII with specific antibodies (phosphorylated and total JNK antibodies are from Cell Signaling, Cat# 4671 and 9252; phosphorylated and total PKCβII antibodies are from Millipore, Cat# 07-873 and 04-406), respectively. Myocyte JNK or PKCβII phosphorylation status was expressed as a ratio of phosphorylated to total JNK or PKCβII protein [14]; [16].

### PKCβ siRNA Transfection

Small-interference RNA (siRNA) specific for PKCβII was purchased from Santa Cruz Biotechnology. Cardiac myocytes were transfected the siRNA using Lipofectamine 2000 reagent (Invitrogen Canada) according to the manufacturer’s instructions [16]. Transfection efficiency was 70% as determined with Western blot and the cardiac myocytes were used in experiments 48 h after the siRNA transfection [14].

### Cardiomyocyte Apoptosis

Cardiomyocyte apoptosis was assessed by determination of caspase 3 activity and fragmented DNA [6] as well as the TUNEL staining. For measuring the caspase 3 activity, the myocytes were washed with PBS and lysed with Cell Lysis Buffer (Biovision). Subsequently, the cell lysates were centrifuged at 10,000 *g* for 10 min at 4°C. The supernatants were incubated with a caspase-3 fluorometric substrate DEVA-AFC (BioVision). The caspase 3 activity was determined by measuring the fluorescence intensity with a Victor 3 multi-label counter (Perkin Elmer).

Apoptotic myocyte death was determined with a quantitative Cell Death Detection ELISA Kit (Roche) to detect fragmented DNA according to manufacturer’s instructions [6]. Briefly, cardiomyocytes were washed with PBS, lysed with cell lysis buffer in the kit and harvested in an Eppendrof tube. After centrifugation at 20,000 *g* for 10 min at 4°C, the supernatants were collected for detection of histone-associated DNA fragments with ELISA.

For TUNEL assay, cardiomyocytes were grown on coverslips, and were washed with PBS and fixed with 4% paraformaldehyde 24 hours post A/R or N/R. Cardiomyocyte apoptosis was detected with TUNEL labeling using an In Situ Cell Death Detection Kit, Fluorescein (Roche) following the manufacturer’s instructions. After TUNEL labeling, nuclei were stained with Hochest 33258, and the TUNEL positive cells were observed using microscope (Zeiss, using 409 objective). The percentage of apoptotic cells per total cells was determined in five randomly chosen fields [14].

### Statistical Analysis

Data are expressed as mean ± SEM. Statistical analysis was performed with two-way ANOVA followed by a Bonferroni correction for multiple comparisons. Graph Pad Software program was used for statistical analysis. A *p* value of less than 0.05 is considered to be statistically significant.

## Results

### IL-33 Attenuates the A/R-induced Cardiomyocyte Apoptosis

**Figure 1:**
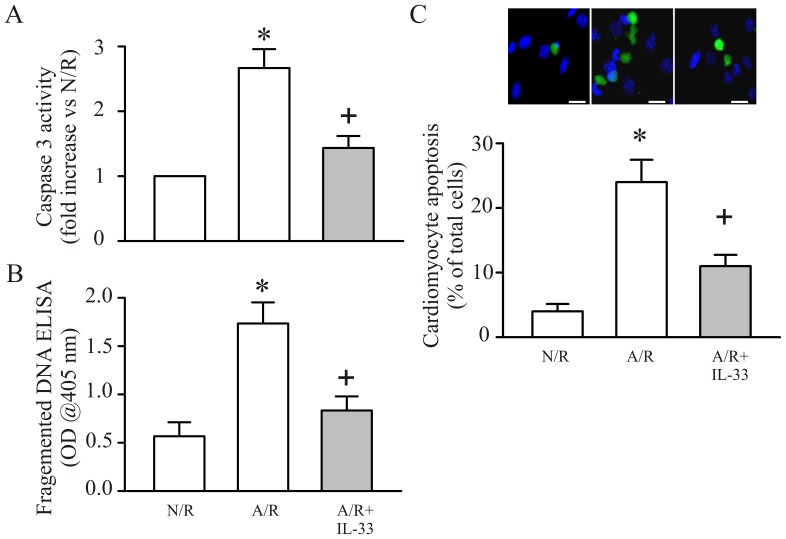
IL-33 attenuates anoxia/reoxygenation (A/R)-induced myocyte apoptosis. Cardiomyocytes were pretreated with IL-33 (5 ng/ml) or vehicle 30 min before A/R. The myocytes were harvested either at 6 hrs or 24 hrs post reoxygenation for caspase 3 activity (**A**) or fragmented DNA ELISA assay (**B**); For some experiments, cardiac myocytes were grown on coverslips challenged with an A/R after IL-33 treatment and detected the myocyte apoptosis with TUNEL staining (**C**). n = 3, *p<0.05 compared with N/R; ^+^p<0.05 compared with A/R.

To examine the effect of IL-33 on cardiomyocyte apoptosis after A/R, the myocyte caspase-3 activity, DNA fragments and TUNEL staining were measured. As shown in **Figure 1**, A/R challenge to cardiomyocytes results in cardiomyocyte apoptosis; the A/R-induced myocyte apoptosis was prevented in the presence of exogenous IL-33 (5 ng/ml).

### IL-33 Prevents the A/R-induced Increase in PKCβ Phosphorylation

**Figure 2:**
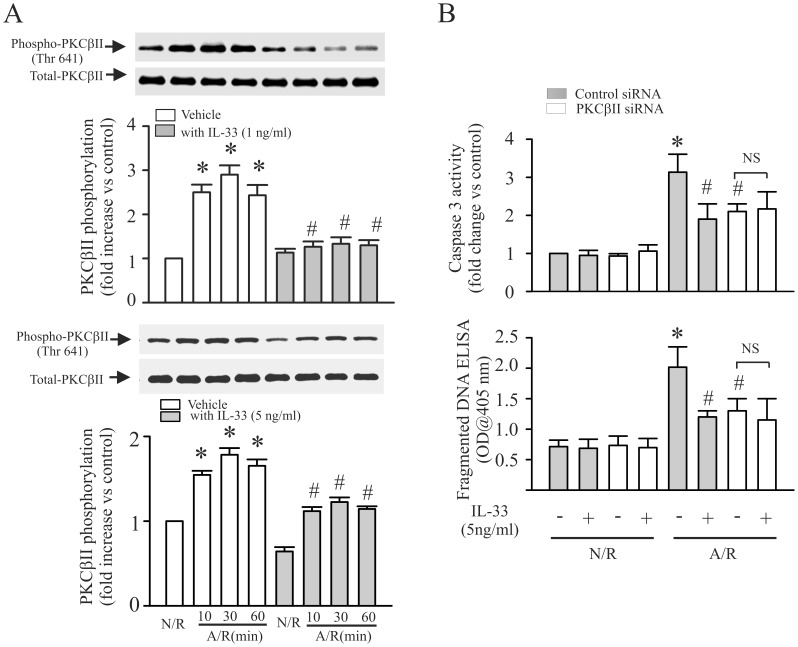
IL-33 attenuates anoxia/reoxygenation (A/R)-induced myocyte apoptosis through inhibition of PKCβ. **A**. Cardiomyocytes were challenged with an A/R with or without IL-33 (1–5 ng/ml) and harvested at the time indicated for PKCβII phosphorylation assay with Western blot. Upper-panel is the actual blots. Lower-panel is the densitometry analysis. n = 3, *p<0.05 compared with N/R without IL-33; ^#^p<0.05 compared with respective A/R without IL-33. **B**. Cardiomyocytes were transfected with siRNA specific to PKCβII, or control siRNA. Subsequently, the myocytes were challenged with an A/R with or without IL-33. The Cardiomycytes were harvested at 6 hrs or 24 hrs after the A/R for caspase 3 or fragmented DNA assay. n = 4, *p<0.05 compared with control siRNA treated myocytes with N/R without IL-33; ^#^p<0.05 compared with control siRNA treated myocytes with A/R without IL-33.

PKCβ activation has been reported contributes to reperfusion-induced lung injury(8). To determine the role of PKCβ on A/R-induced myocyte apoptosis, PKCβII phosphorylation and siRNA knock-down PKCβII were used in the study. As shown in **Figure 2**, A/R challenge to myocytes results in increase in PKCβII phosphorylation (**Figure 2A** **,** open bar). Inhibition of PKCβII with siRNA attenuated the A/R-induced myocyte apoptosis (**Figure 2B**). To determine the role of IL-33 on A/R-induced PKCβII phosphorylation and myocyte apoptosis, cardiomyocytes with or without PKCβII siRNA were treated with IL-33 followed by A/R treatment and PKCβII phosphorylation and myocyte apoptosis assessed. As shown in **Figure 2A** (closed bar), IL-33 (1–5 ng/ml) prevented the A/R-induced PKCβII phosphorylation. In addition, IL-33 (5 ng/ml) did not show protective effect in myocytes with PKCβII siRNA (**Figure 2B**).

### IL-33 Prevents the A/R-induced Increase in JNK Phosphorylation

**Figure 3:**
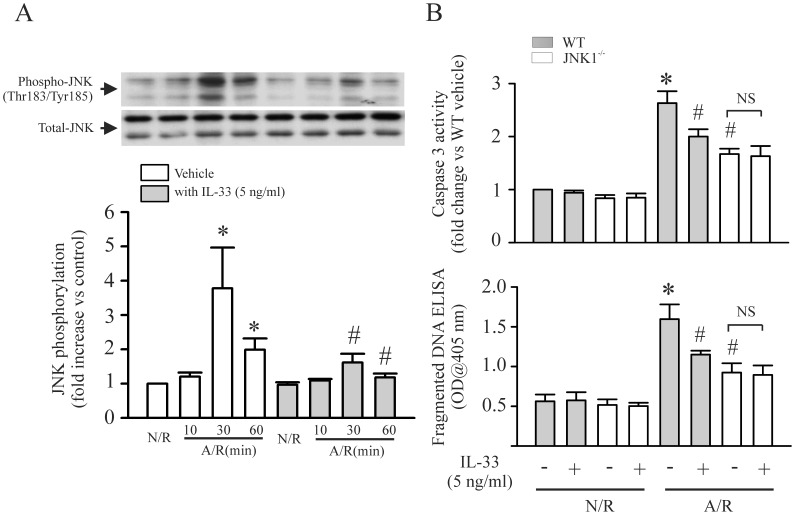
IL-33 attenuates anoxia/reoxygenation (A/R)-induced myocyte apoptosis through inhibition of JNK. **A**. Cardiomyocytes were challenged with an A/R with or without IL-33 and harvested at the time indicated for JNK phosphorylation assay with Western blot. Upper-panel is the actual blots. Lower-panel is the densitometry analysis. n = 3, *p<0.05 compared with N/R without IL-33; ^#^p<0.05 compared with respective A/R without IL-33. **B**. Cardiomyocytes from wild type or JNK^−/−^ mice were challenged with an A/R with or without IL-33. The cardiomycytes were harvested at 6 hrs or 24 hrs after the A/R for caspase 3 or fragmented DNA assay. n = 4, *p<0.05 compared wild type myocytes with N/R without IL-33; ^#^p<0.05 compared with wild type myocytes with A/R without IL-33.

We have previously reported that activation of JNK contributes to A/R-induced cardiomyocyte apoptosis [6]. In the present study, we further confirmed our previous findings. As shown in **Figure 3A** **(**open bar), A/R challenge to myocytes increase in JNK phosphorylation. Genetically inhibition of JNK1 attenuated the A/R-induced myocyte apoptosis (**Figure 3B**). To determine the role of IL-33 on A/R-induced JNK phosphorylation and myocyte apoptosis, cardiomyocytes derived from wild type or JNK1^−/−^ mice were challenged with an A/R with or without IL-33. As shown in **Figure 3A** (closed bar), IL-33 prevented the A/R-induced JNK phosphorylation. In addition, IL-33 did not show protective effect in JNK1^−/−^ cardiomyocytes with A/R (**Figure 3B**).

### Inhibition of A/R-induced PKCβ/JNK Phosphorylation by IL-33 is Dependent on Attenuation of A/R-induced Oxidative Stress in Cardiac Myocytes

**Figure 4:**
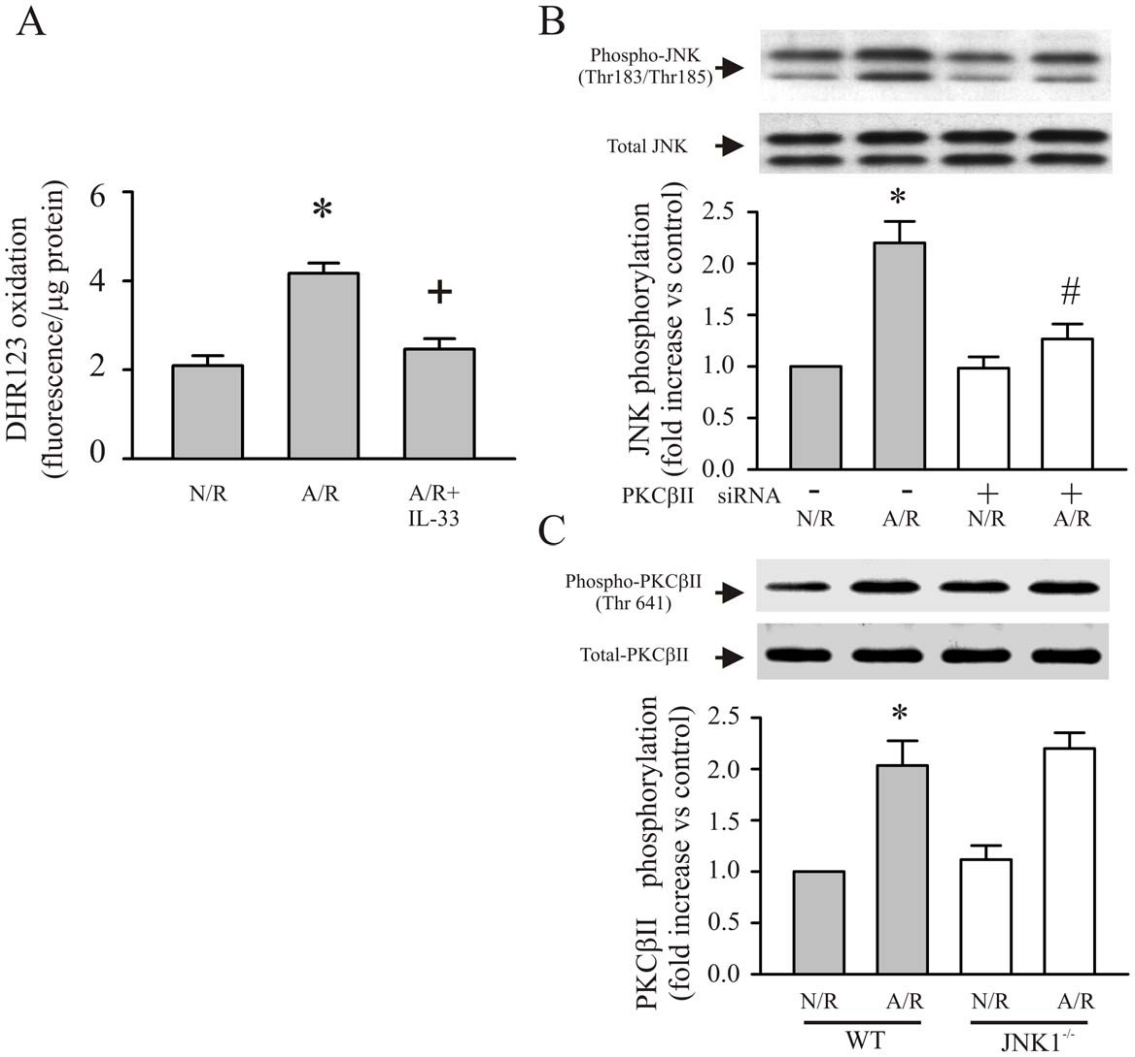
IL-33 prevents the A/R-induced myocyte oxidative stress and inhibition of PKCβ prevented the A/R-induced activation of JNK, while inhibition of JNK showed no effect on the A/R-induced activation of PKCβ. A. Cardiac myocytes were pretreated with IL-33 followed by A/R. Thirty min after the A/R, myocyte oxidant production was assessed by measuring the DHR123 oxidation. n = 3, *p<0.05 compared with N/R, ^+^p<0.05 compared with A/R. B. Cardiac myocytes were transfected with control siRNA or siRNA specific to PKCβII. Subsequently, the myocytes were challenged with an A/R, harvested 30 min after the A/R and JNK phosphorylation assessed with Western blot. n = 3, *p<0.05 compared with control siRNA treated myocytes with N/R (Control); ^#^p<0.05 compared with control siRNA treated cardiomyocytes with A/R. C. Cardiomyocytes derived from wild type or JNK1^−/−^ mice were challenged with an A/R. The myocytes were harvested 30 min after the A/R for detection of PKCβII phosphorylation with Western blot. n = 3, *p<0.05 compared with wild type myocytes with N/R.

PKCβ and JNK have been reported as oxidant sensitive kinases [17]; [18]. Thus, we assessed whether the IL-33 can prevent the A/R-induced myocyte oxidative stress. As shown in Figure 4A, pretreatment of myocytes with IL-33 decreased the myocyte oxidant production after A/R. According to our study, both activation of PKCβ and JNK are pivotal to the A/R-induced myocyte apoptosis. In order to determine whether these two signals can form a signaling pathway after A/R, either phosphorylation of PKCβII or JNK was evaluated after A/R under the inhibition of either JNK1 or PKCβII. As shown in **Figure 4B**, inhibition of PKCβII (siRNA) prevented the A/R-induced JNK phosphorylation. However, genetically inhibition of JNK1 did not show effect on A/R-induced PKCβII phosphorylation (**Figure 4C**). The results indicate that PKCβII phosphorylation is an upstream signal to JNK phosphorylation after A/R.

## Discussion

Abrupt reperfusion of the ischemic myocardium can cause injury to the myocardium. One of the outcomes of the reperfusion-induced injury is myocardial apoptosis [2]; [19]. It is believed that a burst of reactive oxygen species (ROS) will occur on the start of reperfusion which can cause oxidative stress to the myocardium [20]. The oxidative stress can further trigger the intracellular signaling cascades which will lead to induction of cytokine production and myocardial apoptosis [17]; [21]; [22].

PKC family members play integral roles as signal transducers of cell stress response. PKCβ is a conventional form of the PKC [7]. Chronically activation of PKCβ has been reported involved on myocardial dysfunctions related to diabetic mellitus, such as myocardial fibrosis [23]. Previous study has also demonstrated that high level of PKCβ causes myocardial injury [24]. In a mouse model of I/R-induced myocardial injury, Kong L et al demonstrated that PKCβ activation mediates I/R injury as PKCβ deletion or inhibition provide protection to myocardium from I/R-injury [8]. Based on A/R challenge to cardiomyocytes resulted in PKCβII activation and pretreatment of cardiomyocytes with PKCβII siRNA attenuated the A/R-induced myocyte apoptosis [14], results of our *in vitro* study are in agree with previous *in vivo* findings indicating the involvement of PKCβ in the I/R-induced myocardial injury.

JNK is a stress activated protein kinase (SAPK) belongs to mitogen-activated protein kinase family [17]; [25]. The role of JNK in I/R-induced myocardial injury has been extensively studied. Current consensus hold is that activation of JNK in response to oxidative stress post myocardial reperfusion will promote activation of intracellular caspase cascade and lead to myocardial apoptosis [17]. However, the up-stream signal of the JNK after I/R are not fully understood. It is generally believed that JNKs are activated in response to environmental stress or membrane-bound receptor signaling through GTPase of the Rho family through the MAPK kinase kinases. These MAPK kinase kinases then promote activation of the MAPK kinase such as MKK4 and MKK7, which function as dual specificity protein kinases to directly phosphorylate JNKs [17]. In our study, we found A/R challenge to cardiomyocytes resulted in both activation (phosphorylation) of PKCβ and JNK, and inhibition of either PKCβ or JNK attenuated the A/R-induced myocyte apoptosis. In addition, inhibition of PKCβ prevented the A/R-induced JNK activation. The results support that PKCβ/JNK forms a pathway after the A/R challenge and contributes to the A/R-induced myocyte injury.

It is believed that PKCβ and JNK are sensitive to intracellular oxidant stress(17; 18). We and others have previously reported that IL-33 plays protective effect on myocardium in the setting of I/R [13]; [14]. However, the detailed mechanism involved in the protection is not clear. We tested whether IL-33 can directly protect cardiomyocytes from the A/R-induced oxidative stress. The Figure 4A demonstrated that treatment of cardiomyocytes with IL-33 decreased oxidant production after A/R. The results suggested that IL-33 prevents the A/R-induced PKCβ activation through attenuation A/R-induced myocyte oxidative stress. However, how the IL-33 plays its role to attenuate the myocyte oxidative stress is not clear. Further studies are warranted.

It has been demonstrated that IL-33 interacts with its receptor ST-2L and plays antihypertropic effect in the pressure overload-induced myocardial hypertrophy through modulating the NFκB [26]. However, the effect of NFκB on myocardial apoptosis is rather controversial. On the one hand activation of NFκB has been demonstrated to promote apoptosis [27]; on the other hand, increase NFκB activity has been reported to increase the expression of SOD which as anti-apoptotic effect [28]; [29]. In the present study, we demonstrated for the first time the IL-33 plays anti-apoptotic effect through inhibition of PKCβ/JNK pathway.

It has been reported that JNKs are regulated by a multiple-tier module of protein kinase [30]. The canonical upstream kinases contribute to the JNK activation are MAP kinase kinase-4 (MKK4) and MKK7. The MKKs activate JNK by phosphorylate TPY motif (Thr 183 and Tyr 185) of JNK [31]. In our study, although inhibition of PKCβ dramatically attenuated the A/R-induced phosphorylation of JNK on TPY motif, however, it does not completely prevent the JNK phosphorylation (**Figure 4B**). The above observations support that PKCβ is not the only upstream signal of JNK. The phosphorylation of JNK by PKC may amplify the JNK phosphorylation by other kinases (i.e., MKK4/MKK7) as indicated in a study by Lopez-Bergami P and Ronai Z [31]. Whether IL-33 has impact on other upstream kinases of JNK or not remains to be elucidated.

Taken together, in the present study we have demonstrated: 1) PKCβ/JNK, a novel cell signaling pathway is involved in the A/R-induced myocyte apoptosis; 2) IL-33 protects the cardiomyocytes from A/R-induced myocyte apoptosis through inhibition of the PKCβ/JNK pathway. Our study indicates that IL-33 may have therapeutic potential for ischemia/reperfusion-related myocardial injury.
